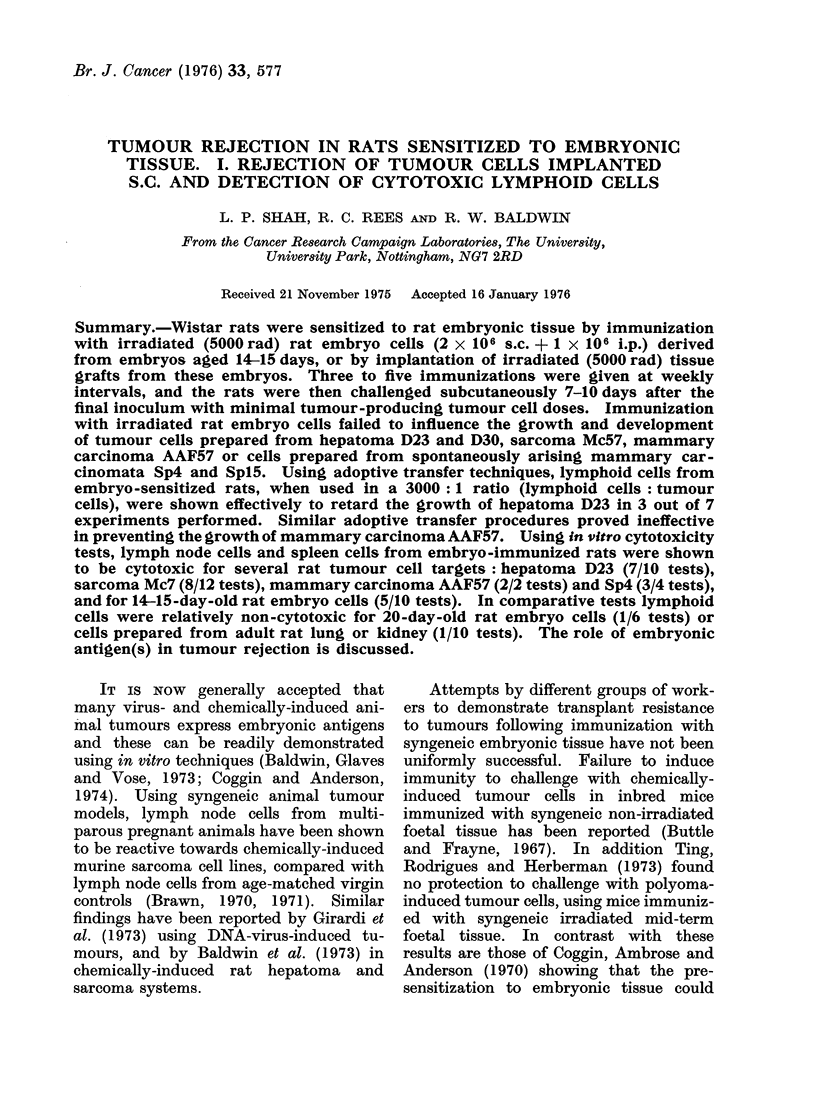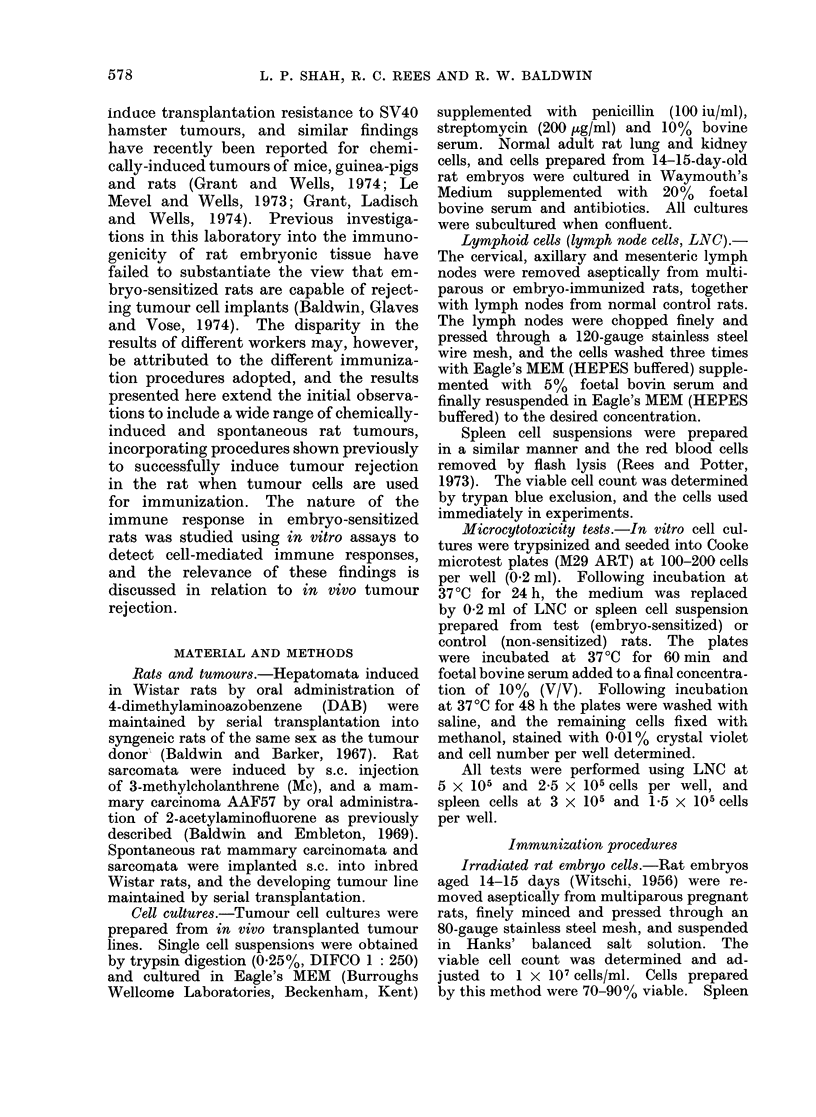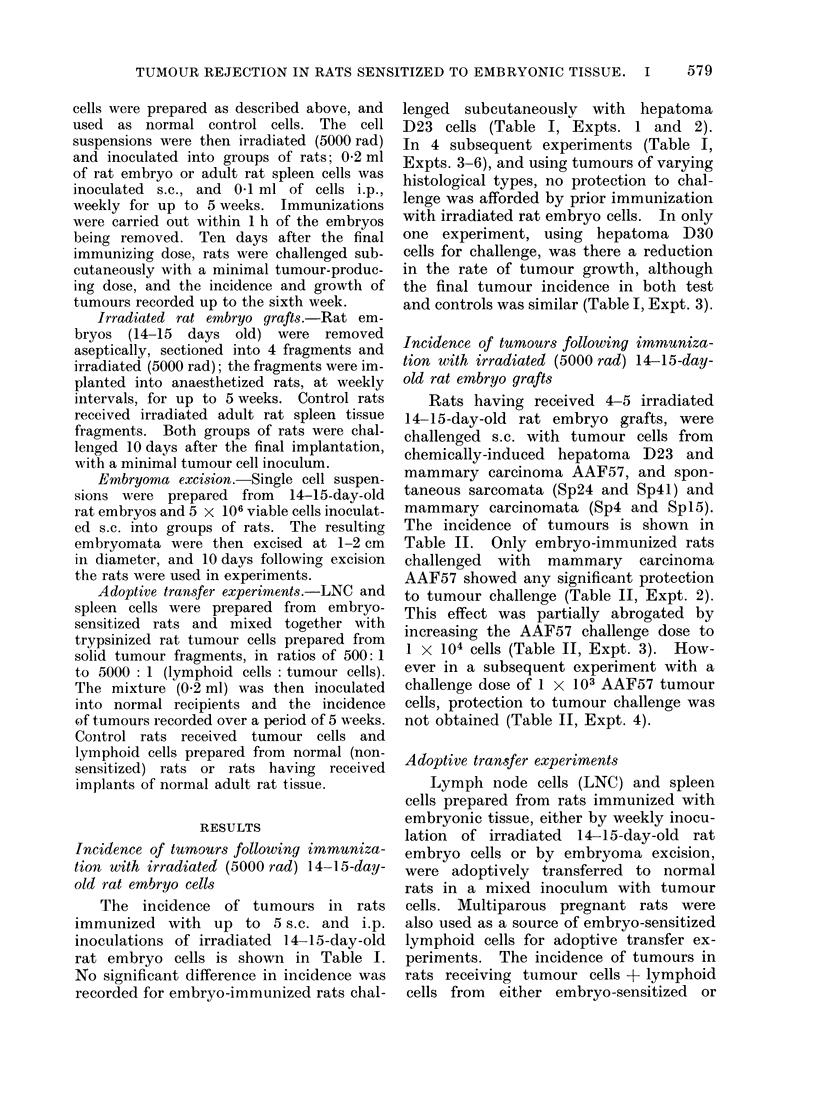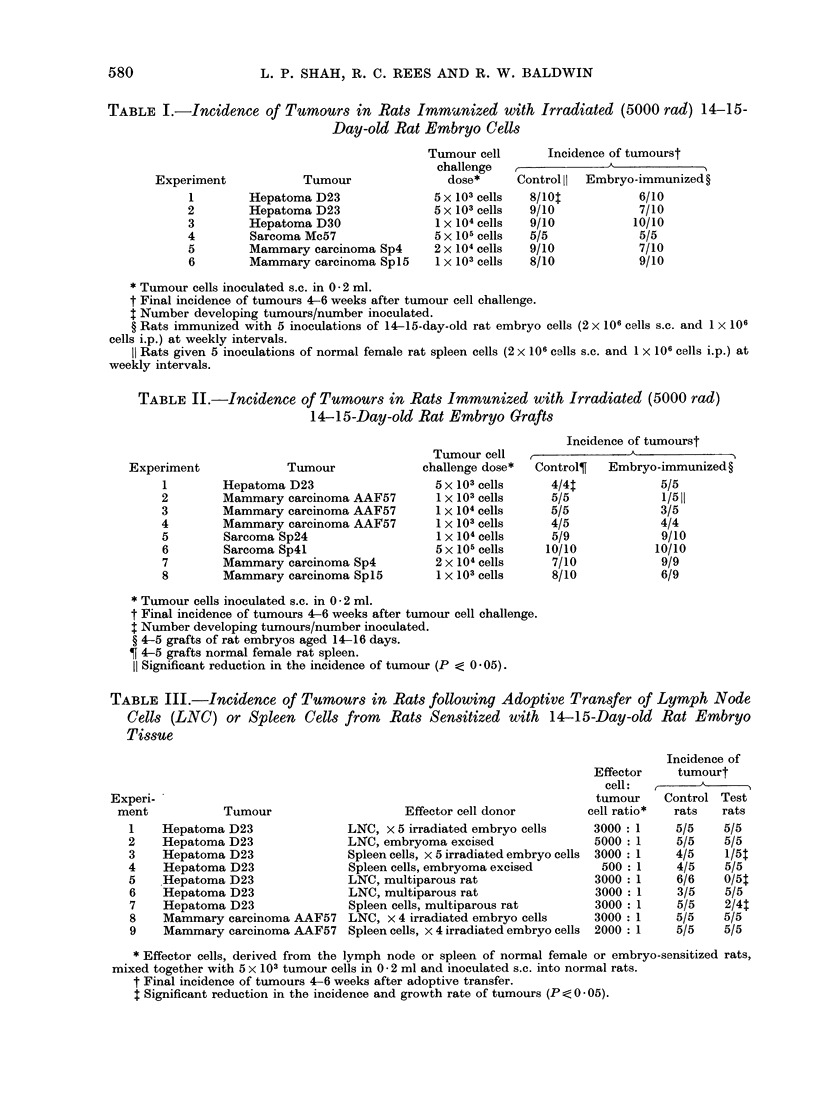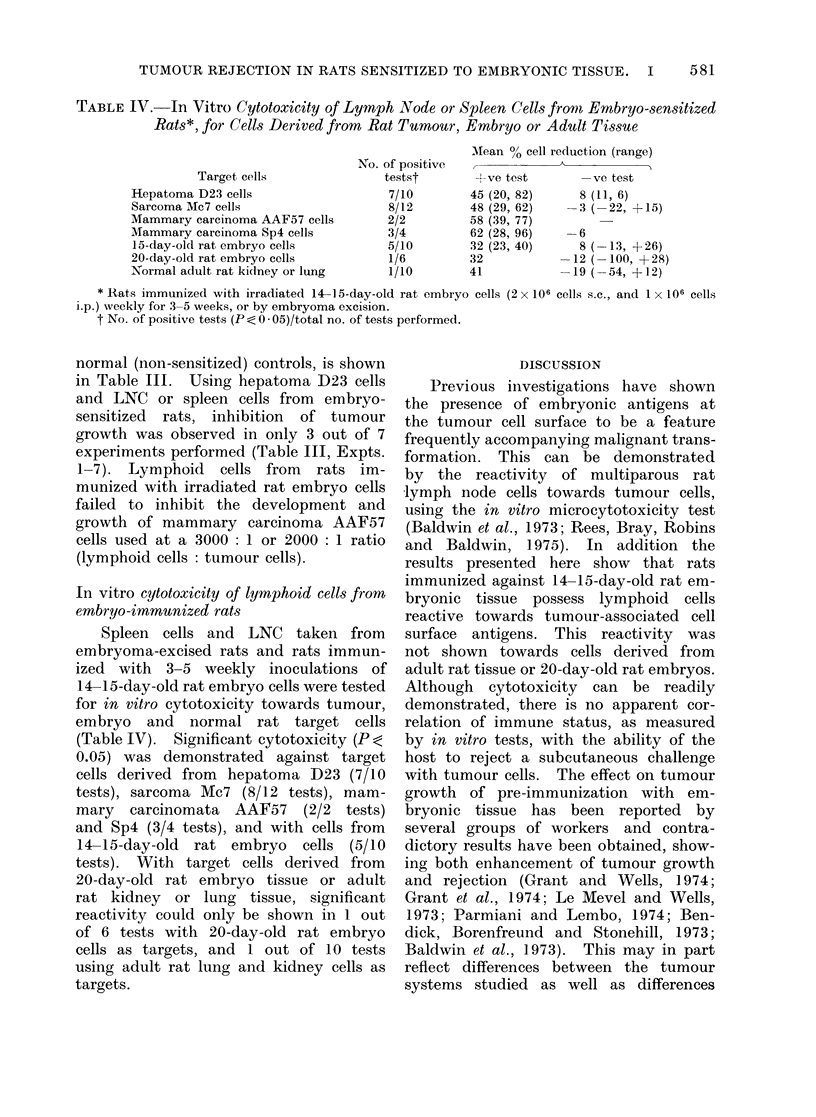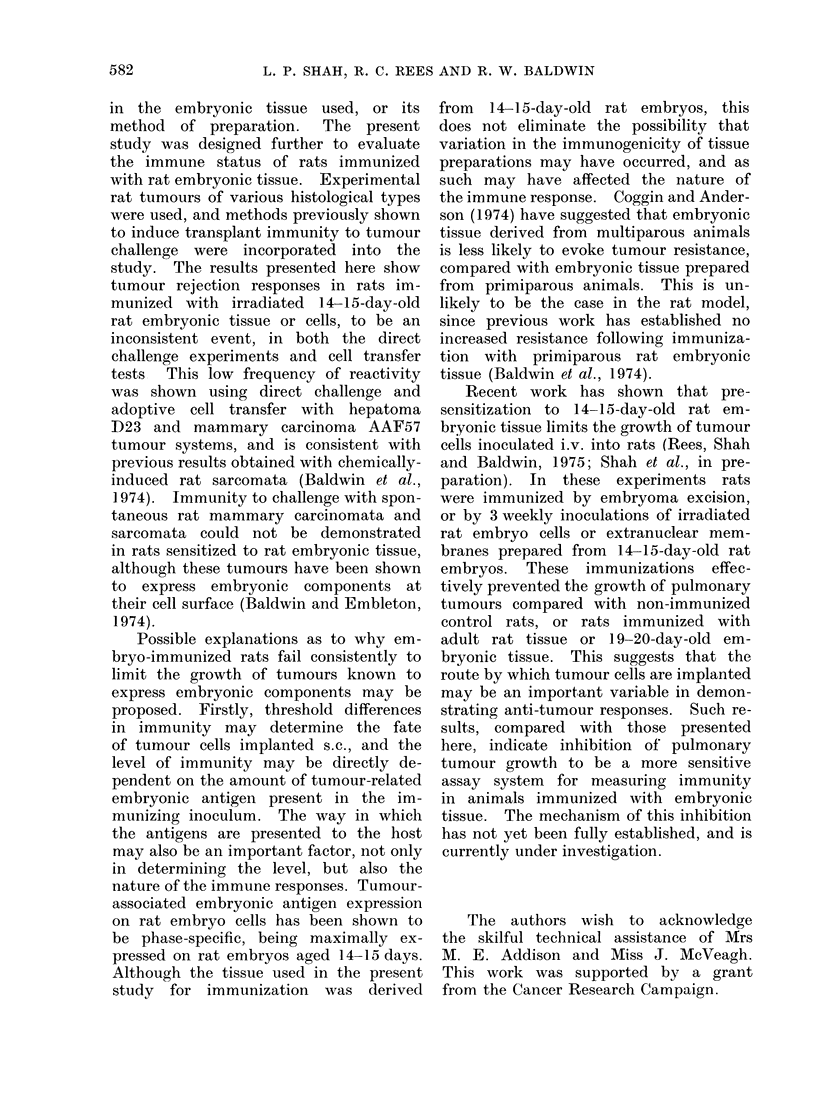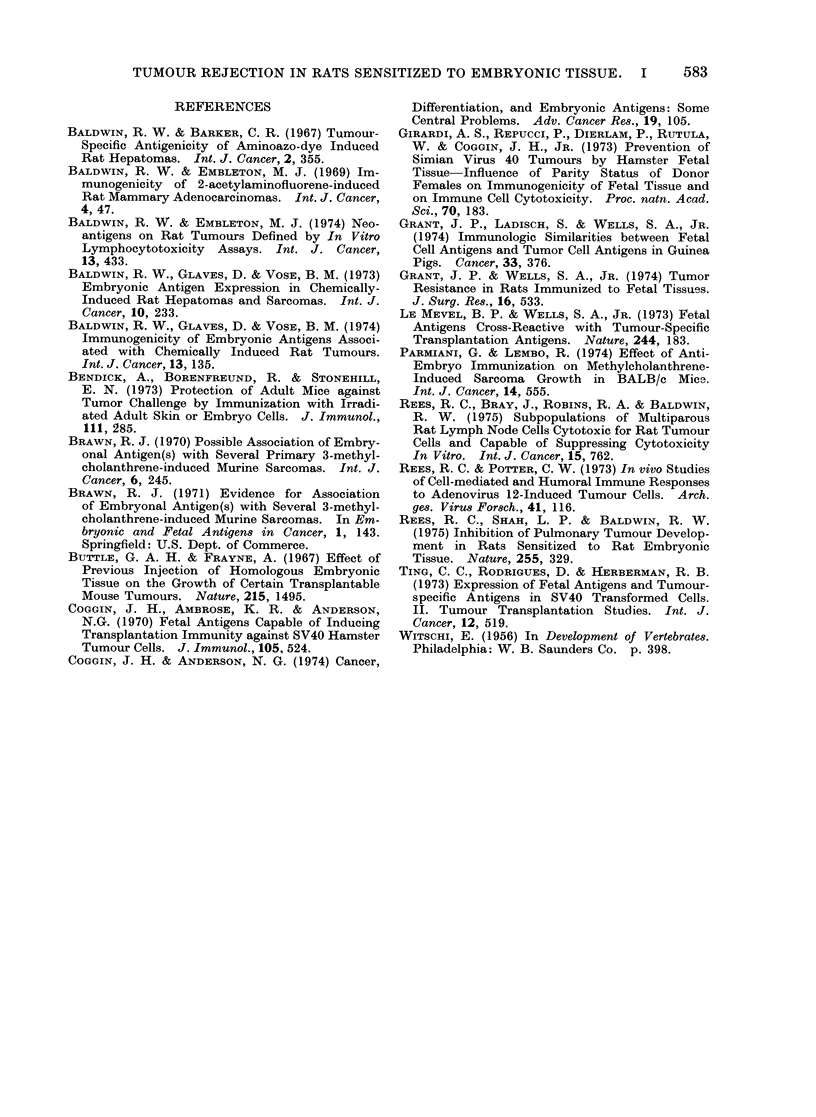# Tumour rejection in rats sensitized to embryonic tissue. I. Rejection of tumour cells implanted s.c. and detection of cytotoxic lymphoid cells.

**DOI:** 10.1038/bjc.1976.94

**Published:** 1976-06

**Authors:** L. P. Shah, R. C. Rees, R. W. Baldwin

## Abstract

Wistar rats were sensitized to rat embryonic tissue by immunization with irradiated (5000 rad) rat embryo cells (2 X 10(6) s.c. + 1 X 10(6) i.p.) derived from embryos aged 14-15 days, or by implantation of irradiated (5000 rad) tissue grafts from these embryos. Three to five immunizations were given at weekly intervals, and the rats were then challenged subcutaneously 7-10 days after the final inoculum with minimal tumour-producing tumour cell doses. Immunization with irradiated rat embryo cells failed to influence the growth and development of tumour cells prepared from hepatoma D23 and D30, sarcoma Mc57, mammary carcinoma AAF57 or cells prepared from spontaneously arising mammary carcinomata Sp4 and Sp15. Using adoptive transfer techniques, lymphoid cells from embryo-sensitized rats, when used in a 3000 : 1 ratio (lymphoid cells : tumour cells), were shown effectively to retard the growth of hepatoma D23 in 3 out of 7 experiments performed. Similar adoptive transfer procedures proved ineffective in preventing the growth of mammary carcinoma AAF57. Using in vitro cytotoxicity tests, lymph node cells and spleen cells from embryo-immunized rats were shown to be cytotoxic for several rat tumour cell targets : hepatoma D23 (7/10 tests), sarcoma Mc7 (8/12 tests), mammary carcinoma AAF57 (2/2 tests) and Sp4 (3/4 tests), and for 14-15-day-old rat embryo cells (5/10 tests). In comparative tests lymphoid cells were relatively non-cytotoxic for 20-day-old rat embryo cells (1/6 tests) or cells prepared from adult rat lung or kidney (1/10 tests). The role of embryonic antigen(s) in tumour rejection is discussed.


*Br. J. Cancer* (1976) **33**, 577

### TUMOUR REJECTION IN RATS SENSITIZED TO EMBRYONIC TISSUE. I. REJECTION OF TUMOUR CELLS IMPLANTED S.C. AND DETECTION OF CYTOTOXIC LYMPHOID CELLS

L. P. SHAH, R. C. REES AND R. W. BALDWIN

*From the Cancer Research Campaign Laboratories, The University, University Park, Nottingham, NG7 2RD*

Received 21 November 1975 Accepted 16 January 1976

**Summary.—Wistar rats were sensitized to rat embryonic tissue by immunization with irradiated (5000 rad) rat embryo cells ($2 \times 10^6$ s.c. $+ 1 \times 10^6$ i.p.) derived from embryos aged 14–15 days, or by implantation of irradiated (5000 rad) tissue grafts from these embryos. Three to five immunizations were given at weekly intervals, and the rats were then challenged subcutaneously 7–10 days after the final inoculum with minimal tumour-producing tumour cell doses. Immunization with irradiated rat embryo cells failed to influence the growth and development of tumour cells prepared from hepatoma D23 and D30, sarcoma Mc57, mammary carcinoma AAF57 or cells prepared from spontaneously arising mammary carcinomata Sp4 and Sp15. Using adoptive transfer techniques, lymphoid cells from embryo-sensitized rats, when used in a 3000 : 1 ratio (lymphoid cells : tumour cells), were shown effectively to retard the growth of hepatoma D23 in 3 out of 7 experiments performed. Similar adoptive transfer procedures proved ineffective in preventing the growth of mammary carcinoma AAF57. Using *in vitro* cytotoxicity tests, lymph node cells and spleen cells from embryo-immunized rats were shown to be cytotoxic for several rat tumour cell targets : hepatoma D23 (7/10 tests), sarcoma Mc7 (8/12 tests), mammary carcinoma AAF57 (2/2 tests) and Sp4 (3/4 tests), and for 14–15-day-old rat embryo cells (5/10 tests). In comparative tests lymphoid cells were relatively non-cytotoxic for 20-day-old rat embryo cells (1/6 tests) or cells prepared from adult rat lung or kidney (1/10 tests). The role of embryonic antigen(s) in tumour rejection is discussed.**

IT IS NOW generally accepted that many virus- and chemically-induced animal tumours express embryonic antigens and these can be readily demonstrated using *in vitro* techniques (Baldwin, Glaves and Vose, 1973; Coggin and Anderson, 1974). Using syngeneic animal tumour models, lymph node cells from multiparous pregnant animals have been shown to be reactive towards chemically-induced murine sarcoma cell lines, compared with lymph node cells from age-matched virgin controls (Brawn, 1970, 1971). Similar findings have been reported by Girardi *et al.* (1973) using DNA-virus-induced tumours, and by Baldwin *et al.* (1973) in chemically-induced rat hepatoma and sarcoma systems.

Attempts by different groups of workers to demonstrate transplant resistance to tumours following immunization with syngeneic embryonic tissue have not been uniformly successful. Failure to induce immunity to challenge with chemically-induced tumour cells in inbred mice immunized with syngeneic non-irradiated foetal tissue has been reported (Buttle and Frayne, 1967). In addition Ting, Rodrigues and Herberman (1973) found no protection to challenge with polyoma-induced tumour cells, using mice immunized with syngeneic irradiated mid-term foetal tissue. In contrast with these results are those of Coggin, Ambrose and Anderson (1970) showing that the presensitization to embryonic tissue could induce transplantation resistance to SV40 hamster tumours, and similar findings have recently been reported for chemically-induced tumours of mice, guinea-pigs and rats (Grant and Wells, 1974; Le Mevel and Wells, 1973; Grant, Ladisch and Wells, 1974). Previous investigations in this laboratory into the immunogenicity of rat embryonic tissue have failed to substantiate the view that embryo-sensitized rats are capable of rejecting tumour cell implants (Baldwin, Glaves and Vose, 1974). The disparity in the results of different workers may, however, be attributed to the different immunization procedures adopted, and the results presented here extend the initial observations to include a wide range of chemically-induced and spontaneous rat tumours, incorporating procedures shown previously to successfully induce tumour rejection in the rat when tumour cells are used for immunization. The nature of the immune response in embryo-sensitized rats was studied using *in vitro* assays to detect cell-mediated immune responses, and the relevance of these findings is discussed in relation to *in vivo* tumour rejection.

### MATERIAL AND METHODS

*Rats and tumours.*—Hepatomata induced in Wistar rats by oral administration of 4-dimethylaminoazobenzene (DAB) were maintained by serial transplantation into syngeneic rats of the same sex as the tumour donor (Baldwin and Barker, 1967). Rat sarcomata were induced by s.c. injection of 3-methylcholanthrene (Mc), and a mammary carcinoma AAF57 by oral administration of 2-acetylaminofluorene as previously described (Baldwin and Embleton, 1969). Spontaneous rat mammary carcinomata and sarcomata were implanted s.c. into inbred Wistar rats, and the developing tumour line maintained by serial transplantation.

*Cell cultures.*—Tumour cell cultures were prepared from *in vivo* transplanted tumour lines. Single cell suspensions were obtained by trypsin digestion (0·25%, DIFCO 1 : 250) and cultured in Eagle's MEM (Burroughs Wellcome Laboratories, Beckenham, Kent) supplemented with penicillin (100 iu/ml), streptomycin (200 $\mu$g/ml) and 10% bovine serum. Normal adult rat lung and kidney cells, and cells prepared from 14–15-day-old rat embryos were cultured in Waymouth's Medium supplemented with 20% foetal bovine serum and antibiotics. All cultures were subcultured when confluent.

*Lymphoid cells (lymph node cells, LNC).*—The cervical, axillary and mesenteric lymph nodes were removed aseptically from multiparous or embryo-immunized rats, together with lymph nodes from normal control rats. The lymph nodes were chopped finely and pressed through a 120-gauge stainless steel wire mesh, and the cells washed three times with Eagle's MEM (HEPES buffered) supplemented with 5% foetal bovin serum and finally resuspended in Eagle's MEM (HEPES buffered) to the desired concentration.

Spleen cell suspensions were prepared in a similar manner and the red blood cells removed by flash lysis (Rees and Potter, 1973). The viable cell count was determined by trypan blue exclusion, and the cells used immediately in experiments.

*Microcytotoxicity tests.*—*In vitro* cell cultures were trypsinized and seeded into Cooke microtest plates (M29 ART) at 100–200 cells per well (0·2 ml). Following incubation at 37 °C for 24 h, the medium was replaced by 0·2 ml of LNC or spleen cell suspension prepared from test (embryo-sensitized) or control (non-sensitized) rats. The plates were incubated at 37 °C for 60 min and foetal bovine serum added to a final concentration of 10% (V/V). Following incubation at 37 °C for 48 h the plates were washed with saline, and the remaining cells fixed with methanol, stained with 0·01% crystal violet and cell number per well determined.

All tests were performed using LNC at $5 \times 10^5$ and $2{\cdot}5 \times 10^5$ cells per well, and spleen cells at $3 \times 10^5$ and $1{\cdot}5 \times 10^5$ cells per well.

### *Immunization procedures*

*Irradiated rat embryo cells.*—Rat embryos aged 14–15 days (Witschi, 1956) were removed aseptically from multiparous pregnant rats, finely minced and pressed through an 80-gauge stainless steel mesh, and suspended in Hanks' balanced salt solution. The viable cell count was determined and adjusted to $1 \times 10^7$ cells/ml. Cells prepared by this method were 70–90% viable. Spleen cells were prepared as described above, and used as normal control cells. The cell suspensions were then irradiated (5000 rad) and inoculated into groups of rats; 0·2 ml of rat embryo or adult rat spleen cells was inoculated s.c., and 0·1 ml of cells i.p., weekly for up to 5 weeks. Immunizations were carried out within 1 h of the embryos being removed. Ten days after the final immunizing dose, rats were challenged subcutaneously with a minimal tumour-producing dose, and the incidence and growth of tumours recorded up to the sixth week.

*Irradiated rat embryo grafts.*—Rat embryos (14–15 days old) were removed aseptically, sectioned into 4 fragments and irradiated (5000 rad); the fragments were implanted into anaesthetized rats, at weekly intervals, for up to 5 weeks. Control rats received irradiated adult rat spleen tissue fragments. Both groups of rats were challenged 10 days after the final implantation, with a minimal tumour cell inoculum.

*Embryoma excision.*—Single cell suspensions were prepared from 14–15-day-old rat embryos and $5 \times 10^6$ viable cells inoculated s.c. into groups of rats. The resulting embryomata were then excised at 1–2 cm in diameter, and 10 days following excision the rats were used in experiments.

*Adoptive transfer experiments.*—LNC and spleen cells were prepared from embryo-sensitized rats and mixed together with trypsinized rat tumour cells prepared from solid tumour fragments, in ratios of 500: 1 to 5000 : 1 (lymphoid cells : tumour cells). The mixture (0·2 ml) was then inoculated into normal recipients and the incidence of tumours recorded over a period of 5 weeks. Control rats received tumour cells and lymphoid cells prepared from normal (non-sensitized) rats or rats having received implants of normal adult rat tissue.

### RESULTS

### *Incidence of tumours following immunization with irradiated* (5000 *rad*) 14–15-*day-old rat embryo cells*

The incidence of tumours in rats immunized with up to 5 s.c. and i.p. inoculations of irradiated 14–15-day-old rat embryo cells is shown in Table I. No significant difference in incidence was recorded for embryo-immunized rats challenged subcutaneously with hepatoma D23 cells (Table I, Expts. 1 and 2). In 4 subsequent experiments (Table I, Expts. 3–6), and using tumours of varying histological types, no protection to challenge was afforded by prior immunization with irradiated rat embryo cells. In only one experiment, using hepatoma D30 cells for challenge, was there a reduction in the rate of tumour growth, although the final tumour incidence in both test and controls was similar (Table I, Expt. 3).

### *Incidence of tumours following immunization with irradiated* (5000 *rad*) 14–15-*day-old rat embryo grafts*

Rats having received 4–5 irradiated 14–15-day-old rat embryo grafts, were challenged s.c. with tumour cells from chemically-induced hepatoma D23 and mammary carcinoma AAF57, and spontaneous sarcomata (Sp24 and Sp41) and mammary carcinomata (Sp4 and Sp15). The incidence of tumours is shown in Table II. Only embryo-immunized rats challenged with mammary carcinoma AAF57 showed any significant protection to tumour challenge (Table II, Expt. 2). This effect was partially abrogated by increasing the AAF57 challenge dose to $1 \times 10^4$ cells (Table II, Expt. 3). However in a subsequent experiment with a challenge dose of $1 \times 10^3$ AAF57 tumour cells, protection to tumour challenge was not obtained (Table II, Expt. 4).

### *Adoptive transfer experiments*

Lymph node cells (LNC) and spleen cells prepared from rats immunized with embryonic tissue, either by weekly inoculation of irradiated 14–15-day-old rat embryo cells or by embryoma excision, were adoptively transferred to normal rats in a mixed inoculum with tumour cells. Multiparous pregnant rats were also used as a source of embryo-sensitized lymphoid cells for adoptive transfer experiments. The incidence of tumours in rats receiving tumour cells + lymphoid cells from either embryo-sensitized or

TABLE I.—*Incidence of Tumours in Rats Immunized with Irradiated* (5000 *rad*) 14–15-*Day-old Rat Embryo Cells*

| Experiment | Tumour | Tumour cell challenge dose* | Incidence of tumours† | |
|---|---|---|---|---|
| | | | Control‖ | Embryo-immunized§ |
| 1 | Hepatoma D23 | $5 \times 10^3$ cells | 8/10‡ | 6/10 |
| 2 | Hepatoma D23 | $5 \times 10^3$ cells | 9/10 | 7/10 |
| 3 | Hepatoma D30 | $1 \times 10^4$ cells | 9/10 | 10/10 |
| 4 | Sarcoma Mc57 | $5 \times 10^5$ cells | 5/5 | 5/5 |
| 5 | Mammary carcinoma Sp4 | $2 \times 10^4$ cells | 9/10 | 7/10 |
| 6 | Mammary carcinoma Sp15 | $1 \times 10^3$ cells | 8/10 | 9/10 |

* Tumour cells inoculated s.c. in 0·2 ml.
† Final incidence of tumours 4–6 weeks after tumour cell challenge.
‡ Number developing tumours/number inoculated.
§ Rats immunized with 5 inoculations of 14–15-day-old rat embryo cells ($2 \times 10^6$ cells s.c. and $1 \times 10^6$ cells i.p.) at weekly intervals.
‖ Rats given 5 inoculations of normal female rat spleen cells ($2 \times 10^6$ cells s.c. and $1 \times 10^6$ cells i.p.) at weekly intervals.

TABLE II.—*Incidence of Tumours in Rats Immunized with Irradiated* (5000 *rad*) 14–15-*Day-old Rat Embryo Grafts*

| Experiment | Tumour | Tumour cell challenge dose* | Incidence of tumours† | |
|---|---|---|---|---|
| | | | Control¶ | Embryo-immunized§ |
| 1 | Hepatoma D23 | $5 \times 10^3$ cells | 4/4‡ | 5/5 |
| 2 | Mammary carcinoma AAF57 | $1 \times 10^3$ cells | 5/5 | 1/5‖ |
| 3 | Mammary carcinoma AAF57 | $1 \times 10^4$ cells | 5/5 | 3/5 |
| 4 | Mammary carcinoma AAF57 | $1 \times 10^3$ cells | 4/5 | 4/4 |
| 5 | Sarcoma Sp24 | $1 \times 10^4$ cells | 5/9 | 9/10 |
| 6 | Sarcoma Sp41 | $5 \times 10^5$ cells | 10/10 | 10/10 |
| 7 | Mammary carcinoma Sp4 | $2 \times 10^4$ cells | 7/10 | 9/9 |
| 8 | Mammary carcinoma Sp15 | $1 \times 10^3$ cells | 8/10 | 6/9 |

* Tumour cells inoculated s.c. in 0·2 ml.
† Final incidence of tumours 4–6 weeks after tumour cell challenge.
‡ Number developing tumours/number inoculated.
§ 4–5 grafts of rat embryos aged 14–16 days.
¶ 4–5 grafts normal female rat spleen.
‖ Significant reduction in the incidence of tumour ($P \leqslant 0 \cdot 05$).

TABLE III.—*Incidence of Tumours in Rats following Adoptive Transfer of Lymph Node Cells* (*LNC*) *or Spleen Cells from Rats Sensitized with* 14–15-*Day-old Rat Embryo Tissue*

| Experiment | Tumour | Effector cell donor | Effector cell: tumour cell ratio* | Incidence of tumour† | |
|---|---|---|---|---|---|
| | | | | Control rats | Test rats |
| 1 | Hepatoma D23 | LNC, ×5 irradiated embryo cells | 3000 : 1 | 5/5 | 5/5 |
| 2 | Hepatoma D23 | LNC, embryoma excised | 5000 : 1 | 5/5 | 5/5 |
| 3 | Hepatoma D23 | Spleen cells, ×5 irradiated embryo cells | 3000 : 1 | 4/5 | 1/5‡ |
| 4 | Hepatoma D23 | Spleen cells, embryoma excised | 500 : 1 | 4/5 | 5/5 |
| 5 | Hepatoma D23 | LNC, multiparous rat | 3000 : 1 | 6/6 | 0/5‡ |
| 6 | Hepatoma D23 | LNC, multiparous rat | 3000 : 1 | 3/5 | 5/5 |
| 7 | Hepatoma D23 | Spleen cells, multiparous rat | 3000 : 1 | 5/5 | 2/4‡ |
| 8 | Mammary carcinoma AAF57 | LNC, ×4 irradiated embryo cells | 3000 : 1 | 5/5 | 5/5 |
| 9 | Mammary carcinoma AAF57 | Spleen cells, ×4 irradiated embryo cells | 2000 : 1 | 5/5 | 5/5 |

* Effector cells, derived from the lymph node or spleen of normal female or embryo-sensitized rats, mixed together with $5 \times 10^3$ tumour cells in 0·2 ml and inoculated s.c. into normal rats.
† Final incidence of tumours 4–6 weeks after adoptive transfer.
‡ Significant reduction in the incidence and growth rate of tumours ($P \leqslant 0 \cdot 05$).

TABLE IV.—In Vitro *Cytotoxicity of Lymph Node or Spleen Cells from Embryo-sensitized Rats*, for Cells Derived from Rat Tumour, Embryo or Adult Tissue*

| Target cells | No. of positive tests† | Mean % cell reduction (range) +ve test | Mean % cell reduction (range) −ve test |
|---|---|---|---|
| Hepatoma D23 cells | 7/10 | 45 (20, 82) | 8 (11, 6) |
| Sarcoma Mc7 cells | 8/12 | 48 (29, 62) | −3 (−22, +15) |
| Mammary carcinoma AAF57 cells | 2/2 | 58 (39, 77) | — |
| Mammary carcinoma Sp4 cells | 3/4 | 62 (28, 96) | −6 |
| 15-day-old rat embryo cells | 5/10 | 32 (23, 40) | 8 (−13, +26) |
| 20-day-old rat embryo cells | 1/6 | 32 | −12 (−100, +28) |
| Normal adult rat kidney or lung | 1/10 | 41 | −19 (−54, +12) |

* Rats immunized with irradiated 14–15-day-old rat embryo cells ($2\times10^6$ cells s.c., and $1\times10^6$ cells i.p.) weekly for 3–5 weeks, or by embryoma excision.

† No. of positive tests ($P\leqslant0\cdot05$)/total no. of tests performed.

normal (non-sensitized) controls, is shown in Table III. Using hepatoma D23 cells and LNC or spleen cells from embryo-sensitized rats, inhibition of tumour growth was observed in only 3 out of 7 experiments performed (Table III, Expts. 1–7). Lymphoid cells from rats immunized with irradiated rat embryo cells failed to inhibit the development and growth of mammary carcinoma AAF57 cells used at a 3000 : 1 or 2000 : 1 ratio (lymphoid cells : tumour cells).

### In vitro *cytotoxicity of lymphoid cells from embryo-immunized rats*

Spleen cells and LNC taken from embryoma-excised rats and rats immunized with 3–5 weekly inoculations of 14–15-day-old rat embryo cells were tested for *in vitro* cytotoxicity towards tumour, embryo and normal rat target cells (Table IV). Significant cytotoxicity ($P\leqslant 0.05$) was demonstrated against target cells derived from hepatoma D23 (7/10 tests), sarcoma Mc7 (8/12 tests), mammary carcinomata AAF57 (2/2 tests) and Sp4 (3/4 tests), and with cells from 14–15-day-old rat embryo cells (5/10 tests). With target cells derived from 20-day-old rat embryo tissue or adult rat kidney or lung tissue, significant reactivity could only be shown in 1 out of 6 tests with 20-day-old rat embryo cells as targets, and 1 out of 10 tests using adult rat lung and kidney cells as targets.

### DISCUSSION

Previous investigations have shown the presence of embryonic antigens at the tumour cell surface to be a feature frequently accompanying malignant transformation. This can be demonstrated by the reactivity of multiparous rat lymph node cells towards tumour cells, using the *in vitro* microcytotoxicity test (Baldwin *et al.*, 1973; Rees, Bray, Robins and Baldwin, 1975). In addition the results presented here show that rats immunized against 14–15-day-old rat embryonic tissue possess lymphoid cells reactive towards tumour-associated cell surface antigens. This reactivity was not shown towards cells derived from adult rat tissue or 20-day-old rat embryos. Although cytotoxicity can be readily demonstrated, there is no apparent correlation of immune status, as measured by *in vitro* tests, with the ability of the host to reject a subcutaneous challenge with tumour cells. The effect on tumour growth of pre-immunization with embryonic tissue has been reported by several groups of workers and contradictory results have been obtained, showing both enhancement of tumour growth and rejection (Grant and Wells, 1974; Grant *et al.*, 1974; Le Mevel and Wells, 1973; Parmiani and Lembo, 1974; Bendick, Borenfreund and Stonehill, 1973; Baldwin *et al.*, 1973). This may in part reflect differences between the tumour systems studied as well as differences in the embryonic tissue used, or its method of preparation. The present study was designed further to evaluate the immune status of rats immunized with rat embryonic tissue. Experimental rat tumours of various histological types were used, and methods previously shown to induce transplant immunity to tumour challenge were incorporated into the study. The results presented here show tumour rejection responses in rats immunized with irradiated 14–15-day-old rat embryonic tissue or cells, to be an inconsistent event, in both the direct challenge experiments and cell transfer tests This low frequency of reactivity was shown using direct challenge and adoptive cell transfer with hepatoma D23 and mammary carcinoma AAF57 tumour systems, and is consistent with previous results obtained with chemically-induced rat sarcomata (Baldwin *et al.*, 1974). Immunity to challenge with spontaneous rat mammary carcinomata and sarcomata could not be demonstrated in rats sensitized to rat embryonic tissue, although these tumours have been shown to express embryonic components at their cell surface (Baldwin and Embleton, 1974).

Possible explanations as to why embryo-immunized rats fail consistently to limit the growth of tumours known to express embryonic components may be proposed. Firstly, threshold differences in immunity may determine the fate of tumour cells implanted s.c., and the level of immunity may be directly dependent on the amount of tumour-related embryonic antigen present in the immunizing inoculum. The way in which the antigens are presented to the host may also be an important factor, not only in determining the level, but also the nature of the immune responses. Tumour-associated embryonic antigen expression on rat embryo cells has been shown to be phase-specific, being maximally expressed on rat embryos aged 14–15 days. Although the tissue used in the present study for immunization was derived from 14–15-day-old rat embryos, this does not eliminate the possibility that variation in the immunogenicity of tissue preparations may have occurred, and as such may have affected the nature of the immune response. Coggin and Anderson (1974) have suggested that embryonic tissue derived from multiparous animals is less likely to evoke tumour resistance, compared with embryonic tissue prepared from primiparous animals. This is unlikely to be the case in the rat model, since previous work has established no increased resistance following immunization with primiparous rat embryonic tissue (Baldwin *et al.*, 1974).

Recent work has shown that presensitization to 14–15-day-old rat embryonic tissue limits the growth of tumour cells inoculated i.v. into rats (Rees, Shah and Baldwin, 1975; Shah *et al.*, in preparation). In these experiments rats were immunized by embryoma excision, or by 3 weekly inoculations of irradiated rat embryo cells or extranuclear membranes prepared from 14–15-day-old rat embryos. These immunizations effectively prevented the growth of pulmonary tumours compared with non-immunized control rats, or rats immunized with adult rat tissue or 19–20-day-old embryonic tissue. This suggests that the route by which tumour cells are implanted may be an important variable in demonstrating anti-tumour responses. Such results, compared with those presented here, indicate inhibition of pulmonary tumour growth to be a more sensitive assay system for measuring immunity in animals immunized with embryonic tissue. The mechanism of this inhibition has not yet been fully established, and is currently under investigation.

The authors wish to acknowledge the skilful technical assistance of Mrs M. E. Addison and Miss J. McVeagh. This work was supported by a grant from the Cancer Research Campaign.

### REFERENCES

BALDWIN, R. W. & BARKER, C. R. (1967) Tumour-Specific Antigenicity of Aminoazo-dye Induced Rat Hepatomas. *Int. J. Cancer*, **2**, 355.

BALDWIN, R. W. & EMBLETON, M. J. (1969) Immunogenicity of 2-acetylaminofluorene-induced Rat Mammary Adenocarcinomas. *Int. J. Cancer*, **4**, 47.

BALDWIN, R. W. & EMBLETON, M. J. (1974) Neoantigens on Rat Tumours Defined by *In Vitro* Lymphocytotoxicity Assays. *Int. J. Cancer*, **13**, 433.

BALDWIN, R. W., GLAVES, D. & VOSE, B. M. (1973) Embryonic Antigen Expression in Chemically-Induced Rat Hepatomas and Sarcomas. *Int. J. Cancer*, **10**, 233.

BALDWIN, R. W., GLAVES, D. & VOSE, B. M. (1974) Immunogenicity of Embryonic Antigens Associated with Chemically Induced Rat Tumours. *Int. J. Cancer*, **13**, 135.

BENDICK, A., BORENFREUND, R. & STONEHILL, E. N. (1973) Protection of Adult Mice against Tumor Challenge by Immunization with Irradiated Adult Skin or Embryo Cells. *J. Immunol.*, **111**, 285.

BRAWN, R. J. (1970) Possible Association of Embryonal Antigen(s) with Several Primary 3-methylcholanthrene-induced Murine Sarcomas. *Int. J. Cancer*, **6**, 245.

BRAWN, R. J. (1971) Evidence for Association of Embryonal Antigen(s) with Several 3-methylcholanthrene-induced Murine Sarcomas. In *Embryonic and Fetal Antigens in Cancer*, **1**, 143. Springfield: U.S. Dept. of Commerce.

BUTTLE, G. A. H. & FRAYNE, A. (1967) Effect of Previous Injection of Homologous Embryonic Tissue on the Growth of Certain Transplantable Mouse Tumours. *Nature*, **215**, 1495.

COGGIN, J. H., AMBROSE, K. R. & ANDERSON, N.G. (1970) Fetal Antigens Capable of Inducing Transplantation Immunity against SV40 Hamster Tumour Cells. *J. Immunol.*, **105**, 524.

COGGIN, J. H. & ANDERSON, N. G. (1974) Cancer, Differentiation, and Embryonic Antigens: Some Central Problems. *Adv. Cancer Res.*, **19**, 105.

GIRARDI, A. S., REPUCCI, P., DIERLAM, P., RUTULA, W. & COGGIN, J. H., JR. (1973) Prevention of Simian Virus 40 Tumours by Hamster Fetal Tissue—Influence of Parity Status of Donor Females on Immunogenicity of Fetal Tissue and on Immune Cell Cytotoxicity. *Proc. natn. Acad. Sci.*, **70**, 183.

GRANT, J. P., LADISCH, S. & WELLS, S. A., JR. (1974) Immunologic Similarities between Fetal Cell Antigens and Tumor Cell Antigens in Guinea Pigs. *Cancer*, **33**, 376.

GRANT, J. P. & WELLS, S. A., JR. (1974) Tumor Resistance in Rats Immunized to Fetal Tissues. *J. Surg. Res.*, **16**, 533.

LE MEVEL, B. P. & WELLS, S. A., JR. (1973) Fetal Antigens Cross-Reactive with Tumour-Specific Transplantation Antigens. *Nature*, **244**, 183.

PARMIANI, G. & LEMBO, R. (1974) Effect of Anti-Embryo Immunization on Methylcholanthrene-Induced Sarcoma Growth in BALB/c Mice. *Int. J. Cancer*, **14**, 555.

REES, R. C., BRAY, J., ROBINS, R. A. & BALDWIN, R. W. (1975) Subpopulations of Multiparous Rat Lymph Node Cells Cytotoxic for Rat Tumour Cells and Capable of Suppressing Cytotoxicity *In Vitro*. *Int. J. Cancer*, **15**, 762.

REES, R. C. & POTTER, C. W. (1973) *In vivo* Studies of Cell-mediated and Humoral Immune Responses to Adenovirus 12-Induced Tumour Cells. *Arch. ges. Virus Forsch.*, **41**, 116.

REES, R. C., SHAH, L. P. & BALDWIN, R. W. (1975) Inhibition of Pulmonary Tumour Development in Rats Sensitized to Rat Embryonic Tissue. *Nature*, **255**, 329.

TING, C. C., RODRIGUES, D. & HERBERMAN, R. B. (1973) Expression of Fetal Antigens and Tumour-specific Antigens in SV40 Transformed Cells. II. Tumour Transplantation Studies. *Int. J. Cancer*, **12**, 519.

WITSCHI, E. (1956) In *Development of Vertebrates*. Philadelphia: W. B. Saunders Co. p. 398.